# Clinical and experimental insight into pathophysiology, comorbidity and therapy of absence seizures

**DOI:** 10.1093/brain/awaa072

**Published:** 2020-05-21

**Authors:** Vincenzo Crunelli, Magor L Lőrincz, Cian McCafferty, Régis C Lambert, Nathalie Leresche, Giuseppe Di Giovanni, François David

**Affiliations:** a1 Department of Physiology and Biochemistry, Faculty of Medicine and Surgery, University of Malta, Msida, Malta; a2 Neuroscience Division, School of Bioscience, Cardiff University, Museum Avenue, Cardiff, UK; a3 Department of Physiology, Faculty of Medicine, University of Szeged, Szeged, Hungary; a4 Department of Physiology, Anatomy and Neuroscience, Faculty of Science and Informatics, University of Szeged, Szeged, Hungary; a5 Department of Anatomy and Neuroscience, University College Cork, Cork, Ireland; a6 Sorbonne Université, CNRS, INSERM, Neuroscience Paris Seine and Institut de Biologie Paris Seine (NPS - IBPS), Paris, France; a7 Cerebral dynamics, learning and plasticity, Integrative Neuroscience and Cognition Center - UMR 8002, Paris, France

**Keywords:** cortico-thalamo-cortical loop, basal ganglia, limbic system, attention deficits, anti-absence drugs

## Abstract

Absence seizures in children and teenagers are generally considered relatively benign because of their non-convulsive nature and the large incidence of remittance in early adulthood. Recent studies, however, show that 30% of children with absence seizures are pharmaco-resistant and 60% are affected by severe neuropsychiatric comorbid conditions, including impairments in attention, cognition, memory and mood. In particular, attention deficits can be detected before the epilepsy diagnosis, may persist even when seizures are pharmacologically controlled and are aggravated by valproic acid monotherapy. New functional MRI-magnetoencephalography and functional MRI-EEG studies provide conclusive evidence that changes in blood oxygenation level-dependent signal amplitude and frequency in children with absence seizures can be detected in specific cortical networks at least 1 min before the start of a seizure, spike-wave discharges are not generalized at seizure onset and abnormal cortical network states remain during interictal periods. From a neurobiological perspective, recent electrical recordings and imaging of large neuronal ensembles with single-cell resolution in non-anaesthetized models show that, in contrast to the predominant opinion, cortical mechanisms, rather than an exclusively thalamic rhythmogenesis, are key in driving seizure ictogenesis and determining spike-wave frequency. Though synchronous ictal firing characterizes cortical and thalamic activity at the population level, individual cortico-thalamic and thalamocortical neurons are sparsely recruited to successive seizures and consecutive paroxysmal cycles within a seizure. New evidence strengthens previous findings on the essential role for basal ganglia networks in absence seizures, in particular the ictal increase in firing of substantia nigra GABAergic neurons. Thus, a key feature of thalamic ictogenesis is the powerful increase in the inhibition of thalamocortical neurons that originates at least from two sources, substantia nigra and thalamic reticular nucleus. This undoubtedly provides a major contribution to the ictal decrease in total firing and the ictal increase of T-type calcium channel-mediated burst firing of thalamocortical neurons, though the latter is not essential for seizure expression. Moreover, in some children and animal models with absence seizures, the ictal increase in thalamic inhibition is enhanced by the loss-of-function of the astrocytic GABA transporter GAT-1 that does not necessarily derive from a mutation in its gene. Together, these novel clinical and experimental findings bring about paradigm-shifting views of our understanding of absence seizures and demand careful choice of initial monotherapy and continuous neuropsychiatric evaluation of affected children. These issues are discussed here to focus future clinical and experimental research and help to identify novel therapeutic targets for treating both absence seizures and their comorbidities.

## Teaser

What causes absence seizures and are they really benign? Crunelli *et al.* review the pathophysiology, pharmacotherapy and neuropsychiatric comorbidities of absence seizures, and highlight the key role of cortical, thalamic and basal ganglia mechanisms in driving absence seizure ictogenesis.

## Introduction

**Figure 1:**
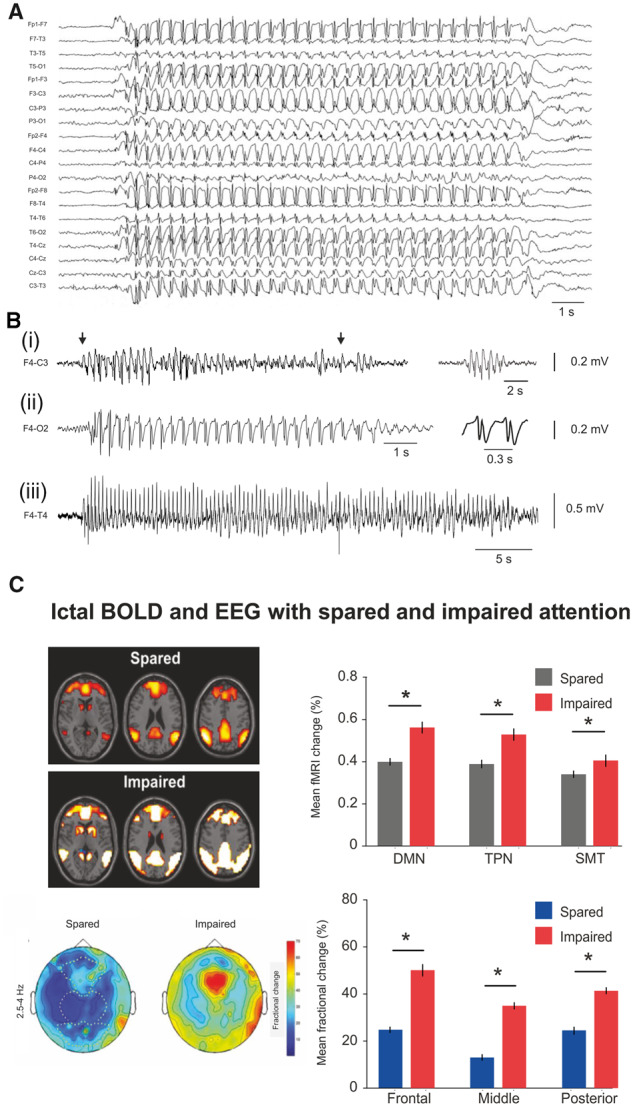
**Spectrum of SWD features and attention levels in children with absence seizures.** (**A**) Characteristic EEG presentation of SWD in CAE. [**B**(**i**)] A long SWD with variable cycle waveform (*left* trace) was associated with lack of consciousness (indicated by the interruption of counting in the period marked by the two arrows) while a short SWD (*right* trace) in the same CAE child was not. [**B**(**ii**)] SWD with double spikes (enlarged on the right). [**B**(**iii**)] A long, hyperventilation-induced SWD from another child with CAE. (**C**) Different absence seizures, characterized by small and large changes in BOLD signal amplitude and 2.5–4 Hz EEG power, are associated with spared and impaired attention, respectively. DMN = default-mode network; TPN = task-positive network; SMT = sensorimotor-thalamic network. **A** is modified from Cerminara *et al.* (2012), and **C** from Guo *et al.* (2016).

Absence seizures are sudden, relatively brief lapses of consciousness associated with lack of voluntary movements and distinctive electrographic spike-wave discharges (SWD) at 2.5–4 Hz (Fig. 1A) (Panayiotopoulos, 1999, 2008; Crunelli and Leresche, 2002*b*; Blumenfeld, 2005*b*; Matricardi *et al.*, 2014). These generalized non-convulsive seizures mostly have a polygenic background and can be present together with other seizure types in various age-dependent and age-independent epilepsies with different rates of remittance and expected outcomes (Crunelli and Leresche, 2002*b*; Camfield and Camfield, 2005; Gardiner, 2005; Fisher *et al.*, 2017; Scheffer *et al.*, 2017). The personal, familial and societal burden of absence seizures is considerable, being influenced by the immediate symptoms of the seizures and the common psychiatric comorbidities, including attentional, cognitive, memory and mood impairments (Masur *et al.*, 2013; Cnaan *et al.*, 2017). Moreover, the high incidence of pharmaco-resistance (Glauser *et al.*, 2013) and the persistence of comorbidities even after full control of the seizures (Masur *et al.*, 2013; Holmes and Noebels, 2016) highlight the need of an improved and holistic mechanistic understanding to facilitate the development of novel medications and other therapeutic approaches that effectively address the entirety of the disease, i.e. seizures and comorbid conditions. Achieving such mechanistic knowledge has undoubtedly been challenging, given the often unrecognized large spectrum of absence seizure semiology and EEG features, the lack of suitable biomarkers, the heterogeneity of (and lack of drug-free) cohorts used in many studies, the ethical restrictions of invasive clinical studies and the less than optimal experimental conditions used in some studies in animal models. Notwithstanding these difficulties and limitations, advancements with significant clinical implications are being made.

Here, we discuss novel insights on absence seizure initiation, ictogenesis, comorbidity and therapy that have originated both from non-invasive human studies and invasive investigations in animal models. Points of controversy and crucial gaps in knowledge will be highlighted raising questions that should contribute to improve current clinical practice and to focus future clinical and experimental research. The emphasis will mainly be on absence seizure of childhood absence epilepsy (CAE) (where these seizures are the only clinical symptom) and on animal models with an absence seizure-only phenotype, as in these children and model systems the epileptogenesis and ictogenesis of absence seizures are not compromised or modulated by the concomitant presence of other types of seizures. Nevertheless, many of the findings, key issues and conclusions raised and discussed in this review do apply to absence seizures that are present in other epilepsies (Guo *et al.*, 2016; Tangwiriyasakul *et al.*, 2018).

### Spectrum of clinical symptoms and electrographic features of absence seizures

Contrary to the classical textbook illustration of regular 2.5–4 Hz SWD (Fig. 1A), the electrographic presentation of absence seizures can be highly variable even in the same individual and may include SWD with different amplitudes and durations, absence of spikes, presence of double spikes and a progressive decrease in frequency towards the end of the paroxysm (Fig. 1B) (Panayiotopoulos, 1999). Moreover, the secondary clinical symptoms of absence seizure (e.g. automatisms, atonic and tonic muscular components, mild clonic eye or mouth jerks etc.) may be highly variable among children and during different absence seizure in the same child. The primary loss of consciousness itself is also variable and a recent study combining functional MRI, EEG and attentional tests has directly demonstrated the broad spectrum of cognitive impairments that is associated with different absence seizures, with some seizures showing a fully spared attention (Fig. 1C) (Guo *et al.*, 2016).

A similarly large spectrum of absence seizure electrographic and behavioural features is also present in various genetic and pharmacological animal models and even in the same mouse or rat during successive absence seizures. However, animal models do show differences with human absence seizures, including a higher SWD frequency at 5–11 Hz and lack of absence seizure remittance with age [for comprehensive summaries of similarities and differences of absence seizures between humans and animal models, see Pitkänen *et al.* (2017)]. In this context, it is important to mention that two recent studies have questioned the validity of current rodent models of absence seizure because (i) these model rats can modify the duration of their SWD (suggesting that they are not adverse events) (Taylor *et al.*, 2017); and (ii) absence seizure-like events are present in wild-caught rats (implying that they are unlikely to represent a pathological trait) (Taylor *et al.*, 2019). Whereas the results of both studies are welcome as they directly confirm and enlarge anecdotal evidence and previous scientific findings made in inbred and outbred rat strains since the 1980s, their interpretations and conclusions do not consider the variability in clinical symptoms and electrophysiological features that are present in human absence seizures.

The issue raised by the first study (Taylor *et al.*, 2017) has been extensively debated previously (Depaulis *et al.*, 2017) and will thus not be addressed further here except for emphasizing that the broad spectrum of clinical symptoms in affected children (and adults) includes absence seizures that are associated with both impaired and unimpaired attention (Fig. 1C) (Guo *et al.*, 2016). Regarding the issue raised by the second study (Taylor *et al.*, 2019), the majority (65%) of SWD recorded by these authors in wild-caught rats are <1 s long (and none >2 s) and would thus be considered to be subclinical seizures, i.e. absence seizures for which the electrographic but not the cognitive and behavioural components may be evident and measurable. Indeed, such short subclinical SWD rarely have an overt clinical correlate and absence seizures with spared attention tend to have a very short duration (∼3 s) (Guo *et al.*, 2016). Nevertheless, the study of Taylor *et al.* (2019) strengthens the view that the potential presence of ethosuximide-sensitive SWD in various ‘normal’ (i.e. non-epileptic) inbred and outbred mouse and rat colonies should always be carefully considered when selecting an appropriate control group for comparison with mouse and rat absence seizure model strains. In other words, animals used as the control group should whenever possible be screened for the presence of absence seizures before inclusion in comparative experiments.

### Absence seizure initiation

The pioneering non-linear analysis of multi-site local field potentials in cortico-thalamic networks carried out by Meeren *et al.* (2002) in a genetic model of absence seizures, the Wistar Albino Glaxo from Rijswijk (WAG/Rij) rats (Depaulis and van Luijtelaar, 2006) showed for the first time the key role of a localized cortical region (the peri-oral region of primary somatosensory cortex) in the initiation of absence seizures. Later work in another genetic model, the Genetic Absence Epilepsy Rats from Strasbourg, GAERS (Depaulis and van Luijtelaar, 2006), characterized the barrel field region of primary somatosensory cortex (Studer *et al.*, 2018) and layer 5/6 excitatory pyramidal neurons (Polack *et al.*, 2007) as the originators of the initial paroxysmal activity. Indeed, absence seizures in GAERS rats are abolished by blocking the firing of these neurons (Polack *et al*., 2009), and are more susceptible to ethosuximide applied in the primary somatosensory cortex than the motor cortex or ventrobasal thalamus (Richards *et al.*, 2003; Manning *et al.*, 2004). Notably, a recent study (Lee *et al.*, 2019) has shown that whereas spontaneous SWD in PLC-β4 knockout mice originate in the somatosensory cortex, the majority of SWD in the pharmacological γ-hydroxybutyric acid (GHB) model (Snead, 1992; Venzi *et al.*, 2015) appear first in the prefrontal cortex, indicating different sites of SWD initiation in different mouse and rat, genetic and pharmacological models as in children with absence seizures (see below).

#### Studies in humans

**Figure 2:**
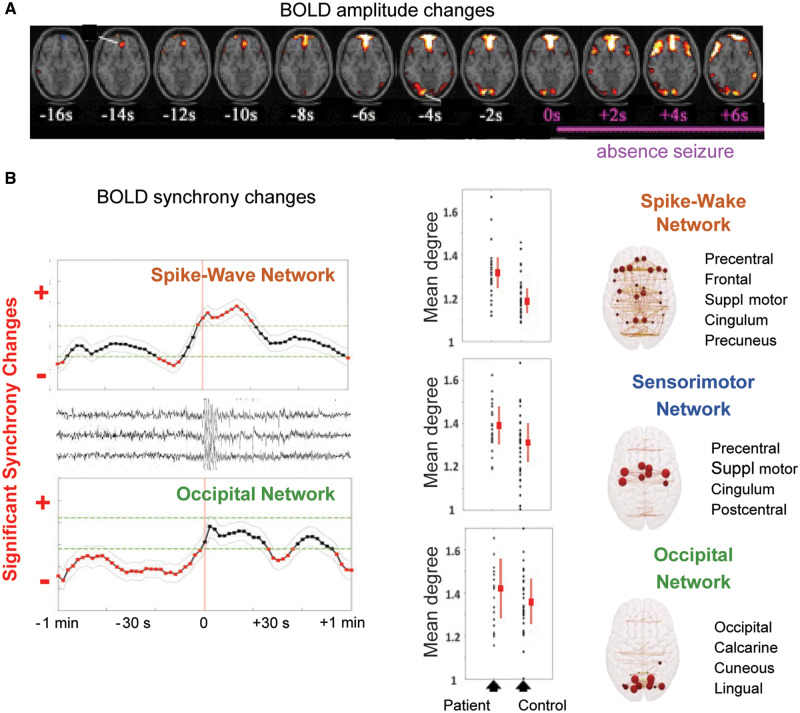
**BOLD signal changes precede absence seizures and persist interictally.** (**A**) Changes in functional MRI BOLD signal amplitude are present in children with absence seizures 14 s before the clinical and electrographic signatures of a seizure are manifested. (**B**) In individuals with generalized SWD, including a small CAE cohort, an increase in the phase-synchrony of functional MRI BOLD signals is present in a ‘spike-wave cortical network’ from a few seconds before until 20 s after a seizure (*top left*) (nodes on the *far right* column). There is also a decreased phase-synchrony in an ‘occipital cortical network’ that starts at least 1 min prior to seizure onset (*bottom left*). Altered phase-synchrony of BOLD signals in the ‘sensorimotor cortical network’ persists interictally but is not present in healthy controls (*middle*). Modified from Bai *et al.* (2010) and Tangwiriyasakul *et al.* (2018).

Blood oxygenation level-dependent (BOLD) functional MRI studies have been critical in identifying key regions involved in human absence seizures (Aghakhani *et al.*, 2004; Gotman *et al.*, 2005; Hamandi *et al.*, 2006; Moeller *et al.*, 2008). In particular, the lack of generalization at the start of an absence seizure has now been confirmed in many multi-modal investigations of young, adolescent and adult populations with absence seizures, including high-density EEG, MEG and functional MRI studies in which localized changes in precuneus, posterior cingulate cortex, lateral parietal cortex and/or frontal cortex are observed before other brain areas become involved in the paroxysm (Holmes *et al.*, 2004; Westmijse *et al.*, 2009; Carney *et al.*, 2010; Bai *et al.*, 2011; Gupta *et al.*, 2011; Benuzzi *et al.*, 2012; Tenney *et al.*, 2013; Wu *et al.*, 2017). Indeed, the first study on a pure CAE cohort reported increases in BOLD signal amplitude occurring from 14 s before the clinical and electrographic signatures of a seizure could be observed (Bai *et al.*, 2010) (Fig. 2A). Notably, the cortical area(s) where these pre-ictal increases in BOLD amplitude occur may differ from child to child but are mostly consistent between seizures for a given child (Bai *et al.*, 2010, 2011; Moeller *et al.*, 2010). Importantly, from absence seizure onset the same cortical areas show a decreased BOLD signal amplitude that may persist for up to 10 s after the end of the seizure (Bai *et al.*, 2010; Moeller *et al.*, 2010; Benuzzi *et al.*, 2012).

Whereas the above functional MRI studies investigated changes in BOLD amplitude, a recent study has analysed the phase-synchrony of BOLD signals in people with generalized SWD, including a small CAE cohort (Tangwiriyasakul *et al.*, 2018). These authors found that there is persistently increased synchrony in a sensorimotor cortical network during non-ictal periods. At least 1 min prior to absence seizure, synchrony in an occipital cortical network begins to decrease (Fig. 2B). Then, from around 10 s prior to SWD onset, the hubs of a SWD-specific cortical network (involving prefrontal regions, precuneus, medial parietal cortex, and medial and lateral prefrontal cortex) begin to show a linear increase in synchrony. Together, this indicates that a persistently abnormal sensorimotor network may represent a ‘pre-ictal’ brain state that can smoothly progress to an ictal SWD network (Fig. 2B). Notably, a high level of synchrony is still apparent in the SWD network 20 s after the end of the electrographic paroxysmal activity, after which the brain reverts to the pre-SWD state (Tangwiriyasakul *et al.*, 2018). In summary, this work demonstrates for the first time the presence of a persistently, i.e. ictally and interictally, altered cortical sensorimotor network in people with absence seizure, a finding of great significance for our understanding of the pathophysiology of these seizures and their comorbidities (see ‘Neuropsychiatric comorbidities of absence seizures’ section).

The existence of altered resting state functional connectivity that may underlie some of the inter-ictal behavioural impairments is supported by the correlation between decreased medial frontal cortex activation during a cognitive task and impaired task performance in CAE (Bai *et al.*, 2011; Killory *et al.*, 2011; Vega *et al.*, 2011; Luo *et al.*, 2014). Furthermore, the variable extent of behavioural impairments during absence seizures has been shown to correlate with the magnitude of ictal BOLD amplitude changes, providing the first indications of where the behavioural component of the seizures might be mediated (Fig. 1B) (Berman *et al.*, 2010; Guo *et al.*, 2016).

#### Studies in animal models

Attempts to gain additional mechanistic insights by comparing non-invasive imaging in humans with simultaneous non-invasive imaging and invasive recordings in animal models have, however, so far been hampered by the fact that rodent models of absence seizures do not show the widespread cortical ictal and post-ictal decreases in multi-modal haemodynamic measurements (functional MRI, cerebral blood flow and volume) (Tenney *et al.*, 2004; David *et al*., 2008; Mishra *et al.*, 2011) as observed in humans (Bai *et al.*, 2010; Moeller *et al.*, 2010; Benuzzi *et al.*, 2012). These conflicting results are not due to the frequency difference between rodent and human SWD (5–11 Hz and 2.5–4 Hz, respectively) as anaesthetized ferrets, which exhibit 3 Hz SWD, show cortical functional MRI increases (Youngblood *et al.*, 2015). This clinical-experimental discrepancy must then be explained by either a mechanistic limitation of absence seizure models, differences in higher cortical function between humans and animals, a peculiarity of human versus lower mammal neurovascular coupling or an effect of the anaesthetics hitherto used in the animal imaging studies. A combination of these factors is also possible: ictal cortical BOLD amplitude changes in awake GAERS rats (Kratochvil *et al.*, 2018) are similar to those in individuals with absence seizures, as are those in Sprague Dawley rats administered the pro-absence drug GHB (Venzi *et al.*, 2015) after recovery from ketamine/medetomidine anaesthesia (Tenney *et al.*, 2003), though WAG/Rij rats still express cortical increases in the same ketamine/medetomidine recovery conditions (Tenney *et al.*, 2004). In summary, given these apparent discrepancies between models (GHB versus WAG/Rij) and drug conditions (GAERS drug-free versus WAG/Rij anaesthetized and WAG/Rij post-anaesthesia), a greater understanding of the case-specific influences on neurovascular coupling (Lemieux *et al.*, 2011; Sieu *et al.*, 2015) is required if any insight into the translational relevance or indeed the mechanisms of absence seizure is to be gained from non-invasive imaging in absence seizure animal models.

#### Significance of new evidence

A number of important implications derive from the findings outlined above.

First, the existence of well-delineated cortical networks or regions where haemodynamic and electrographic changes are first observed before spreading to other areas has raised the question of whether absence seizures should still be considered ‘generalized’ events (van Luijtelaar *et al.*, 2014; Lüttjohann and Van Luijtelaar, 2015). This early pre-ictal cortical activity of absence seizure is a phenomenon very distinct from the structural and functional alterations that characterize the ‘focus’ of focal onset seizures (Fisher *et al.*, 2017; Scheffer *et al.*, 2017), thus emphasizing that the word ‘focus’ should not be used for absence seizures, as has occurred recently (van Luijtelaar and Sitnikova, 2006; Lüttjohann *et al.*, 2011, 2014; Paz and Huguenard, 2015). Rather, in view of the many and far-located areas contributing to the pre-ictal changes of absence seizure, these cortical regions could be accurately referred to as the cortical initiation network (CIN). With this distinction in mind, the current classification of absence seizures as generalized seizures (Fisher *et al.*, 2017; Scheffer *et al.*, 2017) remains clinically appropriate and useful.

Second, larger studies of individuals with absence seizures may disclose different CINs than those so far uncovered (Bai *et al.*, 2010; Tangwiriyasakul *et al.*, 2018) (see also Carney *et al*., 2012; Masterton *et al*., 2013). In turn, these diverse networks may be potentially correlated with different genotypes (Crunelli and Leresche, 2002*b*; Guerrini and Noebels, 2014) and the outcome of monotherapy (Glauser *et al.*, 2013; Shinnar *et al.*, 2017). Indeed, the different location of the pre-ictal changes of cortical BOLD amplitude in various children with absence seizures (Bai *et al.*, 2010, 2011; Moeller *et al.*, 2010) suggests that a diverse genotype may bring about cellular and synaptic alterations preferentially in different cortical networks, a possibility that can be directly tested by large studies of combined functional MRI, EEG and/or MEG of affected individuals with a known genotype.

Third, the existence of a CIN settles the long-standing controversy about cortex or thalamus initiation of absence seizures (Avoli, 2012) in favour of the cortex, but is entirely compatible with the results of many experimental studies since the 1940s showing the emergence of SWD or absence seizures following various means of activation of different thalamic regions in normal (i.e. non-epileptic) animals (Avoli, 2012; Lüttjohann and Van Luijtelaar, 2015; Taylor and Crunelli, 2014; Sorokin *et al*., 2017). That small localized changes in neuronal excitability limited to a restricted cortical or thalamic region can prompt generalized SWD-like activity throughout cortico-thalamo-cortical networks suggests that these networks are naturally susceptible to such paroxysmal oscillations, as indicated by the expression of absence seizures in normal mice following knockout of a single gene (*Cacna1a*, coding for P/Q-type Ca^2+^ channels) selectively in layer 6 pyramidal neurons (Bomben *et al.*, 2016) and the expression of short-duration SWDs in non-epileptic and wild-caught rodents (Taylor *et al.*, 2017, 2019).

Fourth, whereas the above data provide novel insights on absence seizure initiation, there is currently no evidence as to which cortical and thalamic regions (or indeed other brain areas) are crucial for SWD generalization. Many studies in young and adult cohorts with absence seizures indicate the critical involvement of the thalamus in the expression of these seizures (Moeller *et al.*, 2008, 2009, 2010, 2013; Tyvaert *et al.*, 2009; Tenney *et al.*, 2013). Notable in this respect is the observation that in GAERS rats very brief (<1 s), small amplitude SWD that are present in the cortex but not in the thalamus are not accompanied by any overt behavioural sign, i.e. no absence seizure occurs (Polack *et al.*, 2007), suggesting that thalamic recruitment might be a key step for generalization. If so, thalamic higher order and intralaminar nuclei with their wide cortical innervation (Jones, 2003; Giber *et al.*, 2015) might be the critical thalamic region(s). In particular, the rodent thalamic posterior nucleus, roughly equivalent to the anterior pulvinar region in humans (Jones, 2003), may be one of the key hubs for absence seizure generalization in view of (i) its critical involvement in pre- and post-ictal activity (Lüttjohann *et al.*, 2013; Lüttjohann and Van Luijtelaar, 2015; Sysoeva *et al.*, 2016*b*) and at the onset and offset of SWD (Lüttjohann and Pape, 2019); and (ii) the ability of its stimulation to induce a stronger and longer-lasting excitation of cortical layer 2/3 neurons compared to the activation of other thalamic nuclei (Zhang and Bruno, 2019).

### Absence seizure ictogenesis

**Figure 3:**
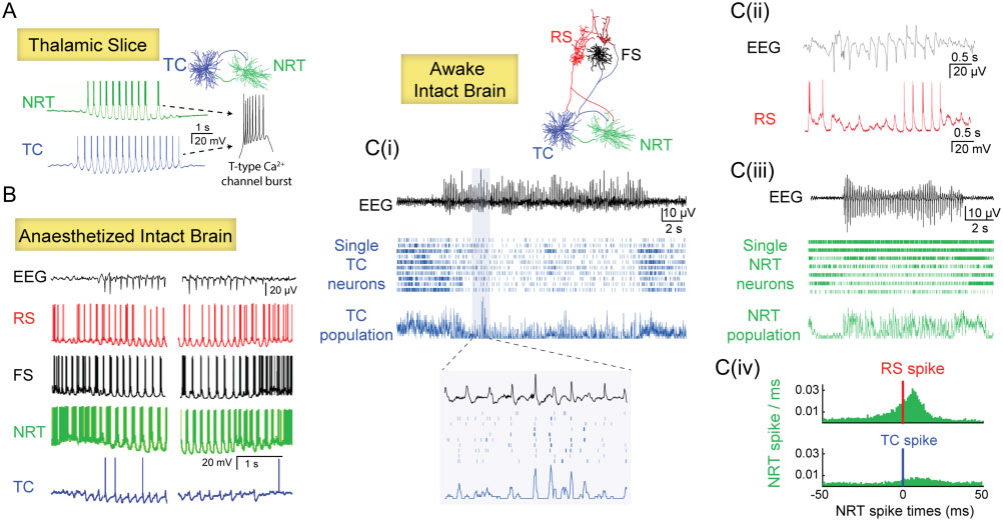
**Absence seizure ictogenesis in cortico-thalamo-cortical networks.** (**A**) In thalamic slices, glutamatergic thalamocortical (TC) neurons and GABAergic reticular thalamic (NRT) neurons elicit a T-type Ca^2+^ channel-mediated burst of action potentials at each cycle of the putative paroxysmal activity. (**B**) In anaesthetized absence seizure models, except TC neurons that fire rarely, all other constituent neurons of cortico-thalamo-cortical networks—cortical regular-spiking excitatory pyramidal neurons (RS), cortical fast-spiking inhibitory neurons (FS) and NRT neurons—show strong (mostly burst) firing at each cycle of a SWD. (**C**) In sharp contrast, in awake animal models, RS neurons [**C**(ii)] show a marked ictal decrease of firing (*bottom trace* is an intracellular recording). The vast majority of TC neurons decrease their firing rate during seizures [raster plot in **C**(i)] though there is a consistent output at each cycle of the SWD when the firing of these neurons is grouped together [see enlarged population activity in **C**(i)]. Two groups of NRT neurons can be distinguished on the basis of their ictal firing: one group that increases their firing ictally (mostly consisting of T-type Ca^2+^ channel-mediated bursts of action potentials) [*top three cells* in the raster plot in **C** (**iii**)] and another group that shows a decreased activity [*bottom three cells* in the raster plot in **C**(**iii**)]. [**C**(**iv**)] Histograms of the activity of NRT neurons recorded simultaneously with an RS (*top*) or and a TC neuron (*bottom*) in a freely moving absence seizure model. Note how, in contrast to results in thalamic slices (**A**), the ictal NRT neuron firing (green bars) is mainly driven by the cortical RS neuron spikes rather than the TC neuron spikes. Time zero marks the firing of the RS and TC neurons (red and blue line, respectively). Modified from Bal *et al.* (1995*b*); Pinault *et al.* (1998); Polack *et al.* (2007); Chipaux *et al.* (2011); McCafferty *et al.* (2018*b*) and Meyer *et al.* (2018).

Our understanding of absence seizure ictogenesis has been dominated for 25 years by the results of brain slice studies, which suggested that hyperexcitability of the glutamatergic thalamocortical and the GABAergic reticular thalamic (NRT) neurons through their bursts of action potentials mediated by T-type Ca^2+^ channels (T-channels) at each paroxysmal cycle of a SWD form the rhythmogenic network mechanism of absence seizures (von Krosigk *et al.*, 1993; Bal *et al.*, 1995*a*,* b*; McCormick and Contreras, 2001) (Fig. 3A). Strong firing at each paroxysmal cycle is also present in NRT neurons and excitatory cortico-thalamic and inhibitory neurons of the CIN in animal models under anaesthesia (with either urethane, ketamine, fentanyl or fentanyl+antipsychotic) (Slaght *et al.*, 2002; Polack *et al.*, 2007; Chipaux *et al.*, 2011; Williams *et al.*, 2016), though under this experimental condition many thalamocortical neurons do not express T-channel-mediated bursts of action potentials and could, in fact, be electrically silent during many paroxysmal cycles within a SWD (Steriade and Contreras, 1995; Pinault *et al.*, 1998, 2006) (Fig. 3B).

#### Recent evidence

Applying electrical recordings and calcium imaging of large neuronal ensembles to freely moving and head-restrained non-anaesthetized animals, respectively, two recent studies (McCafferty *et al.*, 2018*b*; Meyer *et al.*, 2018) have for the first time characterized with single-cell resolution the ictogenic network activity that takes place in cortex and thalamus during absence seizures in three models: a polygenic model (the GAERS rat), a monogenic model (the stargazer mouse) (Noebels *et al.*, 1990) and the pharmacological GHB model in rats.

The first common discovery of both studies is that the vast majority of cortico-thalamic and thalamocortical neurons do not increase their total firing rate during a seizure but exhibit either a decrease or no change, with single neurons of both neuronal populations showing electrical silence at many SWD cycles [Fig. 3C(i, ii, iv) and 4A] (McCafferty *et al.*, 2018*b*; Meyer *et al.*, 2018). When they do fire, they elicit either a single spike or a burst of action potentials in synchrony with the SWD spike.

**Figure 4:**
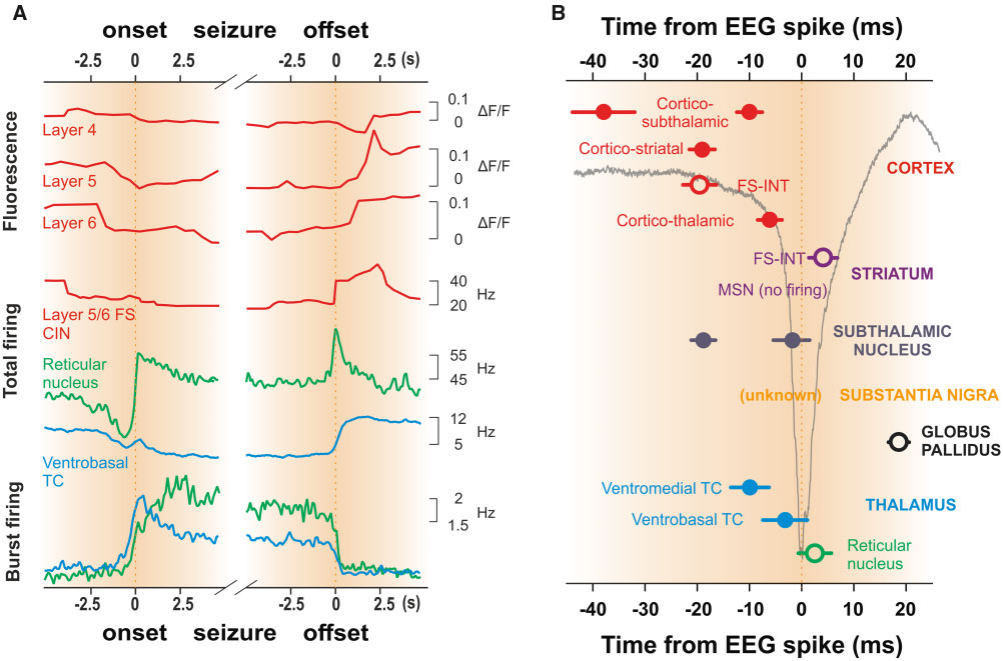
**Pre-ictal and ictal temporal firing dynamics of absence seizures.** (**A**) Average fluorescence changes indicate a decrease in firing in unidentified cortical layer 5 and 6 (but not layer 4) neurons of the visual cortex of non-anaesthetized, head-restrained stargazer mice that begins more than 2 s before and ends almost 2 s after the electrographic seizure. A similar decrease in total firing rate that start 2 s before seizure onset and terminates at seizure offs*et al*so occurs in identified fast-spiking (FS) interneurons of the cortical initiation network (CIN) and in ventrobasal thalamocortical (TC) neurons (blue trace) of freely moving GAERS rats, whereas the total firing GABAergic thalamic reticular neurons (green trace) decreases from 3 s before seizure onset but then show a persistent ictal increase until seizure offset. In contrast, there is an increase in T-channel mediated burst firing in ventrobasal thalamocortical (blue) and thalamic reticular neurons (green) neurons that begins ∼1 s prior to seizure onset and continues until seizure offset. (**B**) Timing of action potential output with respect to the EEG-spike of different neurons in cortico-thalamo-cortical and basal ganglia pathways are superimposed on a schematic spike-and-wave (light black line). Time zero indicates the peak of the EEG spike and individual brain regions are shown on the right. Data for cortico-thalamic CIN layer 5/6, substantia nigra, ventrobasal thalamocortical and reticular nucleus are from freely moving GAERS rats (Deransart *et al.*, 2003; McCafferty *et al.*, 2018*b*), whereas data from striatum, subthalamic nucleus, and globus pallidus are from GAERS rats under neurolept anaesthesia (Slaght *et al.*, 2004; Paz *et al.*, 2005, 2007). As the firing time of cortico-thalamic layer 5/6 neurons in the CIN of freely moving GAERS rats occurs about 10 ms earlier than that of the same GAERS neurons recorded under neurolept anaesthesia, the timing of cortico-subthalamic, cortico-striatal, cortico-thalamic CIN layer 2/3 and layer 4 neurons taken from GAERS under neurolept anaesthesia were modified accordingly. FS-INT = fast-spiking GABAergic interneurons; MSN = striatal medium spiny neurons. Open and filled symbols indicate inhibitory GABAergic and excitatory glutamatergic neurons, respectively. (**A**) Data for CIN FS interneurons are unpublished observations, the others were modified from McCafferty *et al.* (2018*b*) and Meyer *et al.* (2018).

The second key and common finding is that cortico-thalamic, thalamocortical and NRT neurons are not hard-wired to either prevalently express electrical silence, bursts, or single action potential firing but can switch from one pattern to the others in successive paroxysmal cycles and seizures, with some neurons being mostly silent during one seizure but very active in subsequent ones (see Supplementary Fig. 5 in McCafferty *et al.*, 2018*b*) (Meyer *et al.*, 2018). Nevertheless, both cortex and thalamus consistently receive a robust EEG-spike-correlated barrage of action potentials from the other region at each and every SWD cycle see enlarged single cells and population activity in Fig. 3C(i)] (McCafferty *et al.*, 2018*b*), indicating that ictal cortical and thalamic outputs are a population activity, as originally suggested by Buzsáki (1991) for thalamocortical neurons. In this respect, an important finding that has often been overlooked or misinterpreted is that, while the firing in the somatosensory cortex leads that of the somatosensory thalamus in the first 500 ms of a seizure (i.e. in the first three to four paroxysmal cycles in rodents), the activity of thalamic neurons may precede that of cortical neurons or vice versa during subsequent cycles (Meeren *et al.*, 2002): i.e. once a seizure is fully generalized, either of these two regions may, in different paroxysmal cycles within a seizure, take a temporally leading role in reinforcing firing in the other. Thus, the average timing of the ictal firing of cortico-thalamic and thalamocortical neurons (of different thalamic nuclei) within a paroxysmal cycle greatly overlaps, as does that of NRT neurons (Fig. 4B).

The third common and unexpected result is that cortical, thalamocortical and NRT neurons markedly decrease their total firing ∼2–3 s before a SWD can be detected in the EEG (Fig. 4A) (McCafferty *et al.*, 2018*b*; Meyer *et al.*, 2018). In contrast, T-channel-mediated burst firing shows a steep increase that begins 0.5–1.0 s before seizure onset, peaks just few tens of milliseconds and 2.5 s after seizure onset in ventrobasal thalamocortical neurons and NRT neurons, respectively, and is maintained until seizure termination (Fig. 4A) (McCafferty *et al.*, 2018*b*). While at present we do not know the mechanism underlying this pre-ictal reduction in cortical firing, the pre-ictal decrease in thalamocortical and NRT neuron firing might result from the reduced activity of layer 5/6 cortico-thalamic neurons (Fig. 4A). An alternative cause may the increased inhibitory input from a common source, such as the GABAergic neurons of the substantia nigra pars reticulata (SNr), which are known to innervate both NRT neurons and thalamocortical neurons of many first and higher-order thalamic nuclei (Ray and Price, 1992; Miyamoto and Jinnai, 1994; Sakai *et al.*, 1998; François *et al.*, 2002; Cebrián *et al.*, 2005). However, while SNr neurons do markedly increase their firing during absence seizure in freely moving rats (Deransart *et al.*, 2003) (see ‘Essential control of absence seizure by the basal ganglia’ section), it remains to be demonstrated whether such enhancement occurs before seizure onset.

In contrast to cortico-thalamic and thalamocortical neurons, NRT neurons in the intact brain of non-anaesthetized models fire at almost each SWD cycle [Fig. 3C(iii)] and show an increase in their ictal total firing compared to interictal periods (Fig. 4A) (McCafferty *et al.*, 2018*b*). Moreover, in contrast to the results *in vitro* (von Krosigk *et al.*, 1993; Bal *et al.*, 1995*a*,* b*; Huntsman *et al.*, 1999; McCormick and Contreras, 2001; Sohal *et al.*, 2003) and under anaesthesia (Slaght *et al.*, 2002), two populations of NRT neurons can be detected in freely moving models: one that preferentially fires T-channel bursts in successive cycles, while the other fires mostly tonic spikes relatively asynchronously [see top three and bottom four neurons, respectively, in Fig. 3C(iii)], with the former group showing a higher coherence with SWD in the EEG (McCafferty *et al.*, 2018*b*). Whether these two groups may be related to the rostral and caudal sectors of the NRT that have different contributions to absence seizure in genetic models (Aker *et al.*, 2006; Meeren *et al.*, 2009) or to the chemically-distinct populations of NRT neurons that show different bursting activity in normal non-epileptic animals (Halassa *et al.*, 2014) remains to be elucidated. Moreover, in contrast to the prevailing view (von Krosigk *et al.*, 1993; Bal *et al.*, 1995*a*,*b*; Huntsman *et al.*, 1999; McCormick and Contreras, 2001), the ictal firing of NRT neurons in freely moving animals is driven by cortical and not thalamocortical neuron activity [Fig. 3C(iv)]: i.e. the NRT-mediated ictal inhibition of thalamocortical neurons is of a feed-forward and not a feed-back nature.

#### T-type Ca^2+^calcium channels and absence seizures

**Figure 5:**
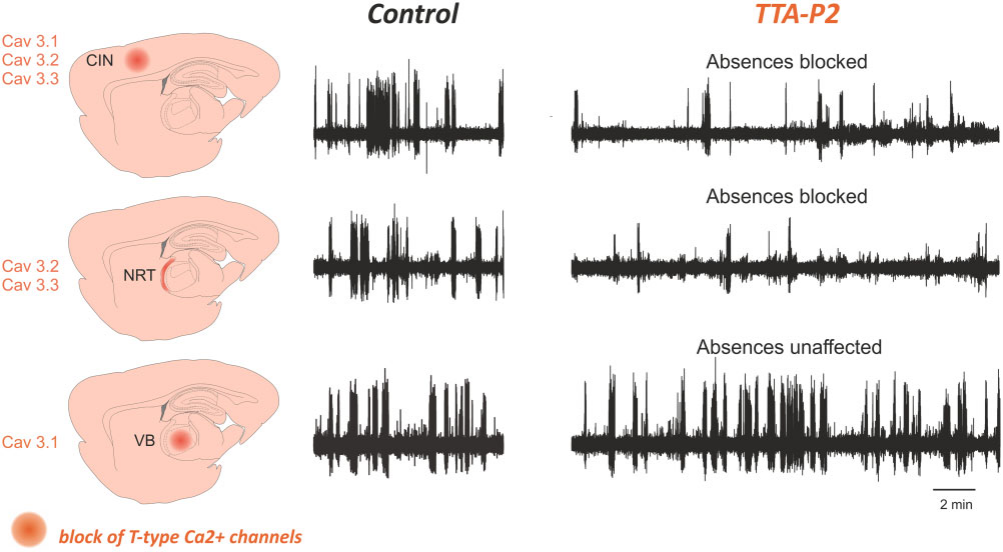
**T-type Ca^2+^ channels in cortex and nucleus reticularis thalami (NRT) are necessary for absence seizures.** Schematic representation of injection sites with their complement of T-channel subtypes (*left*) and original data (*right*) showing the ability of the potent and selective pan T-type Ca^2+^ channel antagonist, TTA-P2, to block absence seizures in GAERS rats when bilaterally injected by microdialysis in the CIN (*top row*) or the NRT (*middle row*). Similar administration in the ventrobasal (VB) thalamic nucleus (one of the somatotopic thalamic nuclei of the CIN in this animal model) that contains only thalamocortical neurons, has no effect on absence seizures (*bottom row*).

Another key finding of McCafferty *et al.* (2018*b*) is that T-channel bursts of cortical and NRT neurons are critically involved in the ictal synaptic interactions with thalamocortical neurons, whereas T-channel bursts of thalamocortical neurons do not play a role in the ictal interactions of these neurons with both cortical and NRT neurons. Indeed, localized injection of TTA-P2, a potent and selective pan-T-channel antagonist (Shipe *et al.*, 2008; Dreyfus *et al.*, 2010), by McCafferty *et al.* (2018*b*) shows that the block of T-channels in the NRT and CIN of GAERS rats drastically decreases absence seizures (see also Avanzini *et al.*, 1992), whereas blocking T-channels in thalamocortical neurons of the ventrobasal thalamus, one of the somatotopic thalamic nuclei of the GAERS CIN, has no effect on absence seizures (Fig. 5), the rhythmicity of the thalamic output and its synchrony with the SWD spike. Thus, the use of TTA-P2 to provide a fast block of T-channel function without compensatory activities shows that T-channels of ventrobasal thalamocortical neurons (i.e. Ca_v_3.1) are not essential for spontaneous absence seizures in GAERS rats whereas those of the CIN (i.e. Ca_v_3.1, Ca_v_3.2 and Ca_v_3.3) and NRT (i.e Ca_v_3.2 and Ca_v_3.3) are (Fig. 5).

This critical role of T-channels of cortical and NRT, but not thalamocortical neurons in experimental absence seizures is compatible with the gain-of-function mutations of the Ca_v_3.2 subtype of T-channels reported in some children with absence seizures (Chen *et al.*, 2003; Khosravani *et al.*, 2004) and in GAERS rats (Talley *et al.*, 2000; Powell *et al.*, 2009; Cain *et al.*, 2018), as this subtype of T-channels is expressed in cortical and NRT neurons but not in thalamocortical neurons (Fig. 5) (Talley *et al.*, 1999). Indeed, selective expression of human Ca_v_3.2 (C456S) mutant channels in the barrel cortex of normal mice does elicit absence seizures (Wang *et al.*, 2015), and *in vivo* knockdown of Ca_v_3.2 in GAERS NRT neurons markedly decreases absence seizures (Cain *et al.*, 2018). Moreover, the lack of an essential role for Ca_v_3.1 subtype of T-channels of ventrobasal thalamocortical neurons in absence seizures of GAERS rats (McCafferty *et al.*, 2018*b*) is compatible with the expression of absence seizure in PLCβ4 knockout mice with increased L-type and Ca_v_3.1 channel function (Cheong *et al.*, 2009). This prior study, in fact, did not  distinguish between L-type and Ca_v_3.1 channels as it used mibefradil, a non-specific blocker of Na^+^, ATP-sensitive K^+^, L-type Ca^2+^ and T-type Ca^2+^ channels (Mishra and Hermsmeyer, 1994; Bezprozvanny and Tsien, 1995; Nilius *et al.*, 1997; Viana *et al.*, 1997; Gomora *et al.*, 1999; Liu *et al.*, 1999).

#### Significance of new evidence

A number of important issues derive from the novel findings outlined above. First, the emerging picture from the investigations conducted in the absence of anaesthesia suggests that, rather than an exclusively thalamic rhythmogenesis, cortical mechanisms are key in driving absence seizures and determining SWD frequency. In particular, the synchronous output of thalamocortical neurons is not determined by the dynamics of their T-channel-mediated burst firing but rather is driven by top-down cortical excitation and sharpened by cortically driven, i.e. feed-forward, NRT inhibition, with both the cortically-driven excitation and the NRT inhibition critically depending on T-channel bursts (McCafferty *et al.*, 2018*b*). Notably, the key role of a cortical drive in absence seizure ictogenesis had been predicted by studies in thalamic slices with an artificially-generated cortical feed-back (Bal *et al.*, 2000; Blumenfeld and McCormick, 2000).

**Figure 6:**
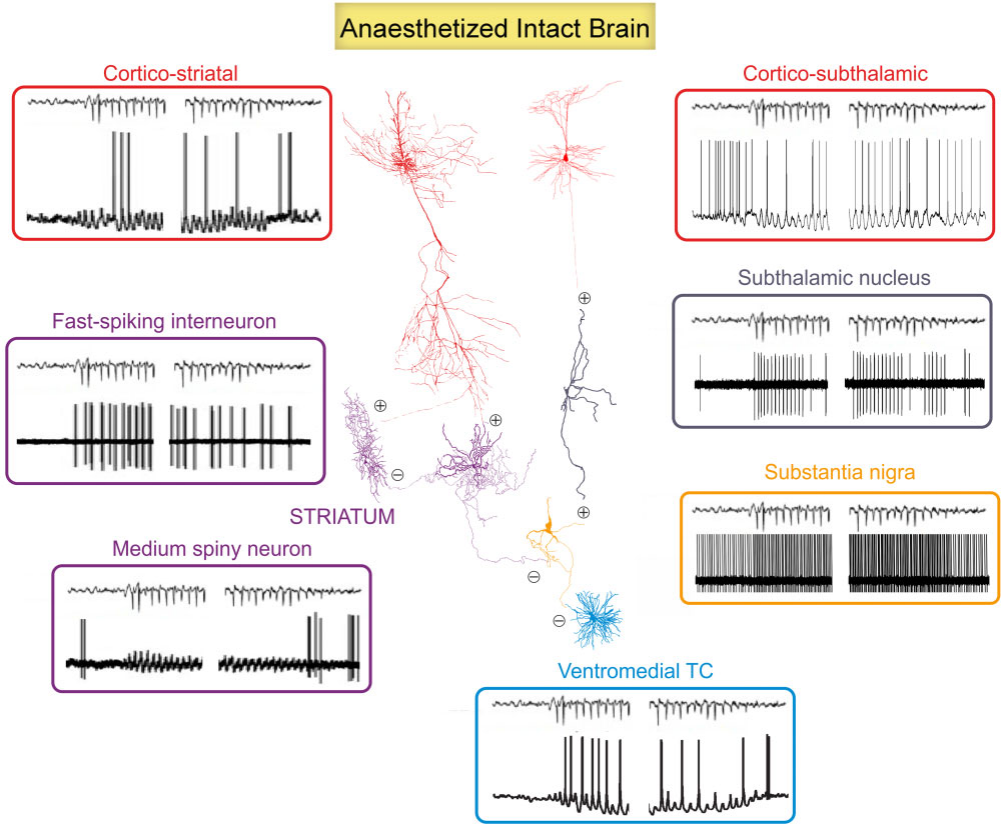
**Ictogenic firing of basal ganglia neurons.** Schematic diagram of the ictal activity of the component neurons of the direct and indirect basal ganglia pathways recorded under neurolept anaesthesia (except the substantia nigra neurons) are illustrated close to the morphological representation of each neuronal type. Electrical activity of fast-spiking striatal interneurons and substantia nigra and subthalamic neurons shows extracellular recordings, the others are intracellular recordings. The excitatory and inhibitory nature of the synaptic connections is indicated by encircled plus and minus symbols. The relative size of the morphological reconstructions has been altered for graphical purposes. See text for additional details. Modified from Deransart *et al.* (2003); Slaght *et al.* (2004) and Paz *et al.* (2005, 2007).

Second, as during absence seizure all thalamic neurons are strongly driven by the excitatory cortical afferent activity, why do thalamocortical neurons show a decrease in ictal firing? The most likely explanation is that the stronger cortico-NRT neuron synapses (compared to cortico-thalamocortical neuron synapses) (Contreras *et al.*, 1993; Golshani *et al.*, 2001) and the robust NMDA-mediated drive of NRT neuron bursts (Lacey *et al.*, 2012; Wang *et al.*, 2015) assure a strong rhythmic inhibition of thalamocortical neurons that overcomes the cortically driven ictal excitation and thus restrict the output of thalamocortical neurons within a narrow temporal window around the EEG spike (Fig. 4B) (McCafferty *et al.*, 2018*b*). Notably, the NRT-mediated inhibition of thalamocortical neurons is strengthened by the increased ictal firing of the GABAergic SNr neurons that is also phase-locked to the SWD-spike (Fig. 6) (see ‘Essential control of absence seizure by the basal ganglia’ section).

Third, the ictal decrease in the correlation of neuronal activity among visual cortex neurons compared to interictal periods in stargazer mice (Meyer *et al.*, 2018) is, on the one hand, surprising, as it leaves unexplained the origin of the EEG spikes that are present in the same cortical region. On the other hand, this decreased correlation may underlie the decreased ictal BOLD signal synchrony observed in the ‘occipital cortical network’ of individuals with generalized SWD and absence seizures (Tangwiriyasakul *et al.*, 2018) (Fig. 2B) and the deficits in visual tasks often reported as comorbid condition of absence seizure (see ‘absence seizure neuropsychiatric comorbidity’ section).

Fourth, the relative paucity of the firing of cortical and thalamic neurons and their variable recruitment between subsequent cycles within a seizure and between seizures in non-anaesthetized animal models is in sharp contrast with the results of *in vitro* studies in cortical and thalamic slices and *in vivo* investigations under anaesthesia (Fig. 3). This conclusively demonstrates that absence seizure ictogenesis is not fully recapitulated either by anaesthetized models (without behaviour, with altered arousal and excitability, and often under the influence of a neuromuscular blocking drug) and by cortical or thalamic slices (in which reciprocal cortico-thalamic and thalamo-cortical connectivity are absent and the membrane potential deviates from that in the awake intact brain). Thus, isolated cortical and thalamic slices are of limited use when investigating the paroxysmal firing dynamics of SWD and care should be taken in the interpretation of firing patterns and temporal dynamics obtained under anaesthetized conditions. Nevertheless, isolated brain slices (from cortex, thalamus or other brain regions) still offer the opportunity to characterize the constitutive biophysical abnormalities of the voltage- and transmitter-gated channels of genetic absence seizure models or transfected human mutant channels.

### Essential control of absence seizures by the basal ganglia

Since the original observation of the anti-absence effect of blocking SNr neuron activity by local injection of muscimol, a GABA-A receptor agonist, in freely moving GAERS rats (Depaulis *et al.*, 1988*a*, *b*), many investigations using localized and selective pharmacological manipulations in this brain region, the striatum and the subthalamic nucleus have demonstrated that both the direct and indirect basal ganglia pathways exert a tight control over absence seizures in different freely behaving genetic and pharmacological models (Depaulis *et al.*, 1989; Deransart *et al.*, 1996, 2000, 2001; Vercueil *et al.*, 1998). Notably, the involvement of the basal ganglia in absence seizures is supported by the consistent finding of ictal changes in functional MRI BOLD signals in the basal ganglia of CAE and adult cohorts with absence seizure (Li *et al.*, 2009; Bai *et al.*, 2010; Berman *et al.*, 2010; Luo *et al.*, 2011).

The patterns and temporal dynamics of the ictal firing of morphologically and electrophysiologically identified neurons of all basal ganglia nuclei have been thoroughly investigated (Slaght *et al.*, 2004; Paz *et al.*, 2005, 2007) and are illustrated in Figs 4B and 6. The ictal activity of cortico-striatal neurons consists of rhythmic depolarizations with mostly single action potential that are phase-locked to the SWD spike. In the striatum, the fast-spiking GABAergic interneurons show short (two to four spikes) high frequency (300–400 Hz) bursts during absence seizures, and thus the striatal output neurons, the GABAergic medium spiny neurons, do not fire ictally but only exhibit large rhythmic subthreshold depolarizations (Slaght *et al.*, 2004). In contrast to cortico-striatal and cortico-thalamic neurons, the cortico-subthalamic neurons show robust firing in two distinct periods, one well before and one around the EEG spike (Paz *et al.*, 2005). This double wave of excitation leads the excitatory subthalamic neurons to have a characteristic temporal profile consisting of a single action potential (well before the SWD spike), which is followed by a short period of silence and then a high frequency burst of action potentials around the SWD spike (Paz *et al.*, 2005). Thus, the output of the basal ganglia to the thalamus, i.e. the firing of the GABAergic SNr neurons, is increased during absence seizures (Deransart *et al.*, 2003) as a result of the combined effect of (i) the ictal electrical silence of the GABAergic striatal medium spiny neurons (Slaght *et al.*, 2004); and (ii) the increased ictal firing of the glutamatergic subthalamic neurons (Fig. 6) (Paz *et al.*, 2005). Notably, all these studies were carried out in GAERS rats and, except the one in the SNr (Deransart *et al.*, 2003), performed under neurolept anaesthesia. Thus, since the firing of both cortico-thalamic, thalamocortical and NRT neurons show subtle but key differences when recorded under this anaesthetic regime and in freely moving animals (see previous section and Fig. 3), it would be important to investigate the activity of the various neuronal populations of the basal ganglia under the latter experimental condition and in other animal models.

A recent study on *STXBP1*, a gene encoding the presynaptic protein Munc18-1, shows that injection of muscimol in the striatum of *Stxbp1*^−/−^ mice blocks their short (0.5–2 s) ethosuximide-sensitive absence seizures (Miyamoto *et al.*, 2019). Moreover, striatal application of NASPM, a selective blocker of calcium-permeable AMPA receptors, which are more abundant in the fast-spiking striatal interneurons than in the striatal medium spiny neurons (Deng *et al.*, 2007), elicits absence seizures in wild-type mice and (continuous) activation of Designer Receptor Exclusively Activated by Designer Drugs (DREADDS)-transfected fast-spiking striatal interneurons markedly reduces ASs in *Stxbp*^+/−^ mice (Miyamoto *et al.*, 2019). These pharmacological and pharmaco-genetic results directly demonstrate a key role for the striatum in the control of absence seizures but are not supported by the lack of changes in the ictal firing of putative striatal fast-spiking and medium spiny neurons. Indeed, the latter result and other findings of Miyamoto *et al.* (2019) are in contrast with previous observations in morphological identified striatal neurons of GAERS rats (see above) (Fig. 6) (Slaght *et al.*, 2004). It could be that different populations of striatal neurons have a differential sensitivity to these drugs because in monkeys there is evidence of a diverse activation of encephalin-, parvalbumin- and substance P-containing striatal neurons of the direct and indirect basal ganglia pathways following different stimulation paradigms of cortico-striatal neurons in the sensorimotor cortex (Parthasarathy and Graybiel, 1997). Thus, further studies using selective manipulations for either the direct or the indirect basal ganglia pathways are required to fully clarify the pro- or anti-absence contribution that the DRD1- and DRD2-containing striatal medium spiny neurons and various striatal interneuron populations may make to absence seizure.

### Enhanced inhibition of thalamocortical neurons: a key ictogenic feature of absence seizures

The essential control of absence seizures by the SNr (Depaulis *et al.*, 1988*a*, *b*, 1989; Deransart *et al.*, 1996, 2001) implies a causal role for the increased firing of its output neurons in absence seizure generation and thus for an enhanced ictal GABAergic inhibition of thalamocortical neurons of the ventromedial, ventrolateral and ventrobasal nuclei as well as the many other thalamic regions, including the limbic thalamus and intralaminar thalamic nuclei, that are innervated by the SNr (Ray and Price, 1992; Miyamoto and Jinnai, 1994; Sakai *et al.*, 1998; François *et al.*, 2002; Cebrián *et al.*, 2005). Notably, recent findings have shown a direct excitatory connection from the substantia nigra to the NRT (Antal *et al.*, 2014), which will undoubtedly add to the ictal cortical excitation of these thalamic GABAergic neurons whose axons are known to cover almost the entire thalamus (Pinault, 2004). Thus, two separate sources of increased ictal GABAergic inhibition, one from the SNr and one from the NRT, engulf thalamocortical neurons of the majority of thalamic nuclei during absence seizure, providing (i) a tight temporal control of the ictal firing of these neurons via activation of synaptic GABA-A receptors, i.e. phasic GABA-A inhibition (Fig. 4B) (McCafferty *et al.*, 2018*b*); and (ii) a persistent decrease in excitability, via activation of extra-synaptic GABA-A receptors, i.e. tonic GABA-A inhibition (Cope *et al.*, 2005). This ictal increase in the inhibition of thalamocortical neurons markedly contributes to the overall decrease in total firing, the paucity of single action potential firing and the increased T-channel-mediated burst firing during absence seizures (Figs 3 and 4A). Within these similarities, however, thalamocortical neurons in the ventrobasal, ventromedial and ventrolateral nuclei do show slightly different ictal firing features (Figs 3 and 6) (Pinault *et al.*, 1998; Paz *et al.*, 2007; McCafferty *et al.*, 2018*b*). Thus, as different classes of layer 5/6 output neurons, i.e. cortico-thalamic, cortico-striatal and cortico-subthalamic neurons, show different ictal firing patterns (Figs 3 and 6), so do thalamocortical neurons in different thalamic nuclei. Though the precise reasons for these differences still remain to be fully elucidated, three possibilities might be considered. First, in contrast to the accepted view the posterior thalamus, but not the ventrobasal nucleus, receives not only inhibitory but also direct excitatory connections from the substantia nigra (Antal *et al.*, 2014). Thus, it may be possible that the balance between SNr-derived inhibition and excitation plays a role in the different ictal firing observed in thalamocortical neurons of the posterior nucleus and other thalamic nuclei. Second, there could be a differential modulation by DRD1 and DRD2 receptors of the tonic GABA-A inhibition in thalamocortical neurons of different thalamic nuclei (Connelly *et al.*, 2013*a*, *b*; Yague *et al.*, 2013). Third, the involvement of GABA-B receptors, including the generation of GABA-B receptor-mediated synaptic potentials (Crunelli *et al.*, 1988; Crunelli and Leresche, 1991; Charpier *et al.*, 1999), their direct modulation of extra-synaptic GABA-A receptors (Connelly *et al.*, 2013*a*, *b*), the regulation of transmitter release via presynaptic GABA-B receptors (Gervasi *et al.*, 2003; Bessaih *et al.*, 2006) and the direct activation of GABA-B receptors of thalamic astrocytes (Gould *et al.*, 2014), may be different in various thalamic regions.

Notably, in some mouse and rat genetic models of absence seizure, including the GAERS rats and the stargazer mice, the combined NRT- and SNr-derived ictal increase in GABAergic inhibition of thalamocortical neurons may be enhanced by the loss-of-function of the astrocytic GABA transporter GAT-1 (but not GAT-3) (Cope *et al.*, 2009; Pirttimaki *et al.*, 2013), providing additional restraints to the thalamocortical neuron firing. Notably, since there is only a silent mutation in *SLC6A1* (the gene encoding GAT-1) in these two absence seizure models (Cope *et al.*, 2009), their GAT-1 loss-of-function might be due to its aberrant intracellular transport, misplaced location around the synaptic cleft, alteration in phosphorylation states or inefficient modulation by various endogenous signalling molecules (Beckman *et al.*, 1999; Venderova *et al.*, 2005; Vaz *et al.*, 2011). Both in GAERS rats and stargazer mice, this GAT-1 loss of function leads to constitutively higher GABA levels in the thalamus, but not somatosensory cortex (Richards *et al.*, 1995) and an increased basal tonic GABA-A inhibition in thalamocortical neurons (Cope *et al.*, 2009; Errington *et al.*, 2011*a*). Indeed, enhanced tonic inhibition in ventrobasal thalamocortical neurons is sufficient to elicit absence seizure in normal mice and rats and is necessary for the expression of absence seizure in the abovementioned models as well as in the GHB and other models (Cope *et al.*, 2009; Errington *et al.*, 2011*b*). These results in animal models are corroborated by the following findings in humans: (i) higher GABA levels were observed in ipsilateral thalamus, but not cortex, of a child with atypical unilateral SWD (Leal *et al.*, 2016); (ii) anti-epileptic drugs that increase GABA levels, such as tiagabine and vigabatrin, aggravate or induce absence seizures and are contraindicated in individuals with absence seizures (Perucca *et al.*, 1998; Ettinger *et al.*, 1999; Panayiotopoulos, 1999); (iii) the vast majority of currently known human mutations in *SLC6A1* lead to a GAT-1 loss-of-function (Mattison *et al.*, 2018); and (iv) absence seizures are by far the most common epileptic phenotype in children with *SLC6A1* mutations (Dikow *et al.*, 2014; Johannesen *et al.*, 2018).

In summary, solid experimental and clinical evidence strongly suggest that, in contrast to the classical view that absence seizures are driven by increased excitation and/or decreased inhibition in all brain regions involved in the paroxysmal activity, the ictogenic thalamic mechanism of absence seizure is uniquely shaped by the enhanced GABAergic inhibition of thalamocortical neurons that derives both from intra-thalamic (NRT), and extra-thalamic (SNr) sources. Whether other sources of thalamic GABAergic inhibition (i.e. zona incerta, anterior pretectal nucleus) (Giber *et al.*, 2008) participate in the ictal increase of thalamocortical neuron inhibition is currently unknown. In some absence seizure models and children with absence seizure, this increased ictal inhibition of thalamocortical neurons is enhanced by the loss-of-function of GAT-1, which does not always result from a genetic mutation of this transporter gene. Whether a similarly increased inhibition and GAT-1 loss-of-function also shape the ictogenic mechanisms of excitatory and inhibitory neurons within cortical and basal ganglia networks remains to be established.

### Pharmaco-resistance of absence seizure: recognizing the problem

**Figure 7:**
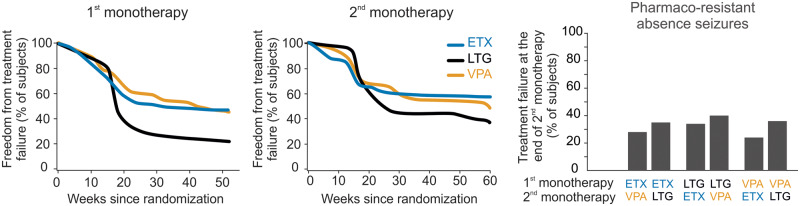
**Pharmaco-resistance of absence seizures.** The first 12-month-long monotherapy with either ethosuximide (ETX) or valproic acid (VPA) is more effective than that with lamotrigine (LTG) in controlling absence seizures (*left*). Similar results are obtained when children unresponsive to the initial monotherapy undergo a second 12-month-long monotherapy with a different drug (*middle*). The combined results of the first and second monotherapy (*right*) show that about 30% of children with childhood absence epilepsy have pharmaco-resistant absence seizures. Modified from Glauser *et al.* (2013) and Cnaan *et al.* (2017).

For many years now, the pharmacological therapy of absence seizures of different epilepsies, and in particular of CAE, juvenile absence epilepsy and juvenile myoclonic epilepsy, has mostly relied on ethosuximide, valproic acid, lamotrigine or any of their combinations (Penovich and Willmore, 2009). In 2013, Glauser *et al.* published the results of the first large (446 newly diagnosed) double-blind randomized controlled trial in a pure CAE cohort that compared the efficacy and tolerability of the above medications. This study shows that at 12 months the freedom-from-failure rate for monotherapy with either ethosuximide or valproic acid is similar (∼45%) and much higher than that for lamotrigine (21%) (Fig. 7). Surprisingly, children who had longer seizures before the start of the pharmacological therapy were more likely to achieve seizure-freedom, a result that did not depend on the type of anti-absence drug used (Dlugos *et al.*, 2013). Notably, children receiving valproic acid performed substantially worse in tests for attention than those receiving ethosuximide or lamotrigine (see below), and 42% of all children who discontinued the treatment because of adverse events were receiving valproic acid. Later, the same authors used their well-characterized large cohort to examine the clinical response to a second monotherapy in children experiencing failure of the initial treatment (Cnaan *et al.*, 2017). After 1 year, 49% of children were seizure-free with a higher proportion receiving ethosuximide or valproic acid (57% or 49%, respectively) than lamotrigine (36%) (Fig. 7). The superior efficacy of ethosuximide over lamotrigine as initial monotherapy and the presence of fewer cognitive and behavioural adverse effects than valproic acid have been confirmed by another recent study (Shinnar *et al.*, 2017) and by analysis of a prospective observational cohort that found better long-term (≥5 and *≥*10 years) outcomes (i.e. seizure- and medication-free) in children initially treated with ethosuximide (76% and 76% complete remission, respectively) than with valproic acid (39% and 44%, respectively) (Berg *et al.*, 2014).

In summary, the serious implications of these studies are that (i) in contrast to prevailing views ‘appropriately chosen and used antiepileptic drugs’ (the ILAE criterion for drug-resistant epilepsy) (Kwan *et al.*, 2009) leave ∼30% of children with uncontrolled absence seizures; and (ii) there are strong neuropsychiatric adverse effects associated with valproic acid treatment, a critical factor to be considered when selecting the first monotherapy in paediatric and juvenile populations with absence seizures.

### Neuropsychiatric comorbidities of absence seizures

#### Studies in humans

**Figure 8:**
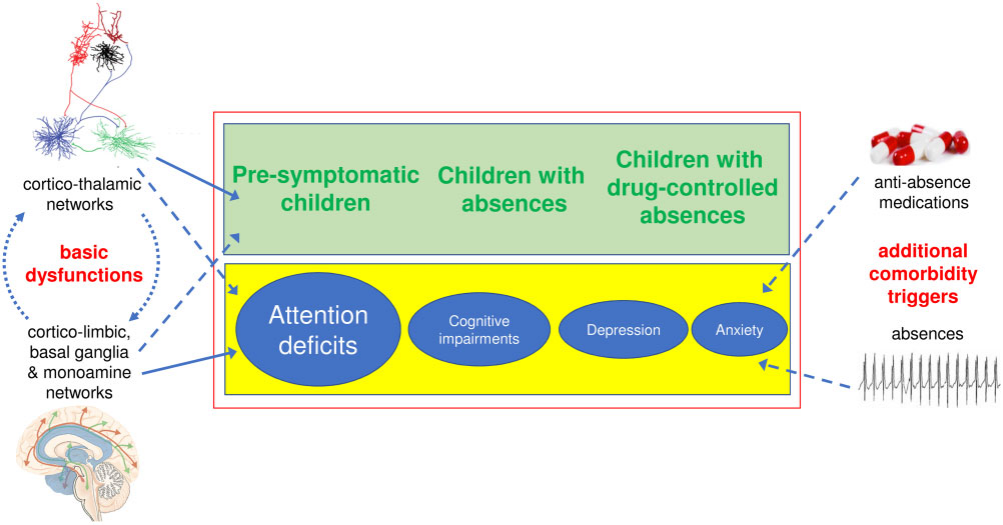
**Neuropsychiatric comorbidities of absence seizures.** Attention deficits are the most common psychiatric comorbidity of childhood absence epilepsy; they can be present in pre-symptomatic children and persist even when seizures are pharmacologically controlled. Other cognitive impairments and mood disorders (e.g. anxiety, depression) may also be present. It is likely that the aberrant cortico-thalamo-cortical networks underlying absence seizure combine with the abnormal basal ganglia-limbic-monoamine networks underlying cognitive impairments and mood disorders to generate the overall neurological and neuropsychiatric phenotype of epilepsies with absence seizures (*left column*). Interactions between these abnormal networks might contribute to a lower seizure threshold and an increased risk of comorbidity. Moreover, absence seizures and anti-absence drugs may induce, or aggravate existing, comorbid conditions (*right column*). Solid and dashed blue lines represent established and putative links, respectively.

It is only relatively recently that the full extent of the psychiatric comorbid conditions that accompany absence seizures has been investigated, though clear warnings were available since the early 1960s from studies of mixed-age mixed-epilepsy populations with generalized SWD, mostly under pharmacological therapy (Hertoft, 1963; Ounsted *et al.*, 1963; Loiseau *et al.*, 1983; Wirrell *et al.*, 1997; Aldenkamp *et al.*, 2001; Ott *et al.*, 2001; Henkin *et al.*, 2005; Vanasse *et al.*, 2005). The unpromising picture that emerges from recent investigations shows that ∼60% of children with CAE have neuropsychiatric comorbidities, with attention deficits as the most common comorbidity (35–40%), followed by mood disorders (Caplan *et al.*, 2008; Glauser *et al.*, 2010; Masur *et al.*, 2013; Gencpinar *et al.*, 2016; Lee *et al.*, 2018) (Fig. 8), in sharp contrast to the high prevalence of depression associated with convulsive seizures (Noebels, 2015*b*). Notably, in the only study of a large (446 children) drug-naïve CAE cohort, the impairments in attention were not of an ADHD type as they were ‘associated more with an inattentive than hyperactive form of attention deficit, i.e. loss of focus on a task more than impulsivity of response’ (Masur *et al.*, 2013), a result similar to that reported previously in a smaller cohort of children with absence seizures and other idiopathic epilepsies (Hermann *et al.*, 2007). Moreover, though not always assessed in drug-naïve populations, reductions of visual-related functions, including visual memory and visuo-spatial tasks, have also been consistently reported (Fedio and Mirsky, 1969; Jambaqué *et al.*, 1993; Pavone *et al.*, 2001; Nolan *et al.*, 2004; Cheng *et al.*, 2017), a finding that may be linked to the recently reported decrease of interictal BOLD synchrony in the occipital cortex of a CAE cohort (Tangwiriyasakul *et al.*, 2018) and the lack of ictal firing synchrony among visual cortical neurons in an absence seizure model (Meyer *et al.*, 2018).

The presence of neuropsychiatric deficits is complemented by structural imaging data in cohorts with absence seizures showing (i) smaller grey matter volume of brain regions involved in attention (including orbital frontal gyrus and temporal lobes) (Caplan *et al.*, 2009); (ii) smaller volume of the amygdala (a region linked to both attention and anxiety/depression) (Schreibman Cohen *et al.*, 2009); (iii) altered age-related changes in cortical thickness and sulcal depth (Tosun *et al.*, 2011); and (iv) diffusion tensor imaging abnormalities in default mode network nodes (Qiu *et al.*, 2016). Functional imaging evidence of abnormalities in the default mode, dorsal attention and salience networks have also been reported (Berman *et al.*, 2010; Killory *et al.*, 2011; Luo *et al.*, 2014; Li *et al.*, 2015). Moreover, BOLD signal synchrony in frontal and precuneus regions is altered ictally while that in precentral areas and cingulum is affected both ictally and interictally (Tangwiriyasakul *et al.*, 2018) (Fig. 2B). However, more than 90% of the children and adults enrolled in all these structural and functional imaging studies were on anti-absence medication for variable lengths of time and no analysis of the drug effects as a covariate was carried out: related confounds cannot, therefore, be discounted since the effect of anti-absence drugs on both structural and functional brain imaging features both in humans and animal models is unknown.

Psychiatric comorbid conditions, in particular attention deficits, may precede the first absence seizure and the epilepsy diagnosis (Hermann *et al.*, 2007; Jones *et al.*, 2007) (Fig. 8). Whereas these results may suggest that the pathophysiological abnormalities underlying absence seizure comorbidities are at least partially independent from seizure occurrence, it is highly likely that the epileptogenesis existing in presymptomatic children may also underlie (and/or contribute to) their comorbid conditions (Fig. 8). Moreover, attention deficits do persist even when seizures are pharmacologically controlled (Williams *et al.*, 2002; Glauser *et al.*, 2013; Masur *et al.*, 2013), and notably more children show this comorbidity after valproic acid (49%) than ethosuximide (32%) and lamotrigine (24%) monotherapy (Glauser *et al.*, 2013). However, it remains unclear whether the comorbidity observed after anti-absence drug treatment is pre-existing, drug-induced and/or due to lack of seizure control (Fig. 8, right). The mutual causal interaction between epilepsy and comorbidity is a very complex one in particular for epilepsies with a genetic aetiology (Noebels, 2015*b*; Keezer *et al.*, 2016): answering the above question, therefore, is essential and will require knowledge of the psychiatric effects of anti-absence drugs in healthy individuals (and normal animals, see below), which unfortunately are currently unknown (Loring *et al.*, 2007; Verrotti *et al.*, 2018).

#### Studies in animal models

Absence seizure models do show psychiatric comorbidities, providing additional validity to these models and a means for future mechanistic studies into these conditions. Subtle alterations in attention, that may be task-specific, have been reported in GAERS (Marques-Carneiro *et al.*, 2016; Marks *et al.*, 2016*a*, *b*) and WAG/Rij rats (Karson *et al.*, 2012; Jafarian *et al.*, 2015), but the majority of the animal studies has focused on anxiety‐ and depression-like behaviours that have been consistently observed in GAERS (Jones *et al.*, 2008; Bouilleret *et al.*, 2009, Powell *et al.*, 2009, 2014*a*, *b*; Tringham *et al.*, 2012; Dezsi *et al.*, 2013; Marques-Carneiro *et al.*, 2014) and WAG/Rij rats (Sarkisova *et al.*, 2010; Russo *et al.*, 2011; van Luijtelaar *et al.*, 2013; Moyanova *et al.*, 2018). Notably, anxiety and absence seizures in GAERS rats can be reduced by environmental enrichment and these anxiolytic and anti-epileptogenic effects are heritable into the next generation (Dezsi *et al.*, 2016), prompting future investigations on potential epigenetic contributions to human absence seizures and their comorbidity. Moreover, rearing off-spring of WAG/Rij rats by non-epileptic Wistar mothers from the second postnatal day results in a marked decrease of absence seizures in early adulthood (Sitnikova *et al.*, 2016), suggesting that an increased maternal care and/or a reduction in stressful environment early in development may contribute to alleviate genetically determined absence seizures.

Chronic (19 weeks) and acute administration of ethosuximide mitigate the anxiety-like phenotype in GAERS (Dezsi *et al.*, 2013), and anxiety and depression in SWD-expressing Long-Evans rats (Shaw *et al.*, 2009). In WAG/Rij rats, chronic ethosuximide ameliorates depressive-like behaviour (Sarkisova *et al.*, 2010; van Luijtelaar *et al.*, 2013) (but see Russo *et al.*, 2011). Notably, however, the abnormal phase-amplitude coupling of EEG beta-gamma waves observed in stargazer mice is unaffected by ethosuximide (Maheshwari *et al.*, 2017), indicating that aberrant cortical circuit-level dynamics persist even after seizure control. The pan-T-channel blocker Z944 abolishes absence seizures, reduces aggressive behaviour, rescues both recognition memory deficits (Marks *et al.*, 2016*a*) and sociability impairment (Henbid *et al.*, 2017), but increases anxiety-like behaviour in GAERS rats (Marks *et al.*, 2019). Thus, this potent and selective T-channel blocker controls absence seizures, but in contrast to the weak and non-selective T-channels blocker ethosuximide (Crunelli and Leresche, 2002*a*), it worsens anxiety-like behaviour, at least in GAERS rats. Finally, chronic lamotrigine, which is the least effective absence seizure monotherapy in humans (Glauser *et al.*, 2013; Cnaan *et al.*, 2017), blocks absence seizures and ameliorates comorbid anxiety- and depression-like phenotypes in Long-Evans rats (Huang *et al.*, 2012). Notably, no study has investigated whether anti-absence drugs induce psychiatric deficits in normal non-epileptic (either young or adult) animals, except for one investigation reporting negative effects of ethosuximide on fear memory but not spatial learning (Ponnusamy and Pradhan, 2006).

However, the interpretation of many of these studies on comorbidity in absence seizure models is compromised by several issues. First, the majority of these investigations did not record the EEG during the behavioural tests, making it impossible to ascertain how the observed changes in behaviour are linked to ictal and interictal periods. Second, in studies using inbred absence seizure models, a comparison with normal animals as well as inbred non-epileptic control strains would be essential to control for potential effects of long-term inbreeding of the non-epileptic strain (Marques-Carneiro *et al.*, 2014; Bombardi *et al.*, 2018). Third, all but two studies (Marks *et al.*, 2016*a*; Henbid *et al.*, 2017) have used only male animals. Fourth, fear-reaction tests are not the ideal procedure to study attention and memory in absence seizure models that have inherent anxiety- and depression-like comorbid conditions and should be replaced by touch-screen technology (Markham *et al.*, 1996).

#### Significance of new evidence

In summary, attention deficits are the most common psychiatric comorbidity of absence seizures when present as the only seizure type: they may precede the first absence seizure (i.e. are present in pre-symptomatic children), persist even after seizures are pharmacologically controlled (Fig. 8) and are much more frequent following monotherapy with valproic acid than ethosuximide or lamotrigine. Other cognitive impairments and mood disorders may also be present (Fig. 8), but systematic analyses of their incidence, life-progression and causal link with treatment are not yet available. It is likely that the aberrant function of cortico-thalamo-cortical networks (underlying the generation of SWD and absence seizures) (Crunelli and Leresche, 2002*a*; Blumenfeld, 2005*a*) combines with the abnormal activity of basal ganglia-limbic-monoamine networks (underlying attention deficits, cognitive impairments and mood disorders) (De Deurwaerdère *et al.*, 2017; Miskowiak and Petersen, 2019; Park *et al.*, 2019) to generate the overall neuropsychiatric comorbid phenotype of absence seizure (Fig. 8, left). Notably, anterior and mediodorsal thalamic nuclei, which show consistent ictal BOLD changes in individuals with absence seizure (Tyvaert *et al.*, 2009) and are crucial for attentional control (Schmitt *et al.*, 2017), provide a structural and functional bridge between limbic and cortico-thalamo-cortical networks (Jones, 2003). Moreover, alterations in cognition and memory may be significantly influenced by the characteristic biophysical properties of thalamocortical neurons (Connelly *et al.*, 2015, 2016, 2017) and by the T-channel-mediated low-threshold spike and bursts that occur during absence seizures, as long-term changes in the plasticity of thalamic synapses preferentially occur when thalamic neurons fire T-channel bursts of action potentials and are rarely observed when these neurons fire tonic single action potentials (Sieber *et al.*, 2013; Pigeat *et al.*, 2015; Leresche and Lambert, 2017; Crunelli *et al.*, 2018). Unravelling the true nature of the link between absence seizure epileptogenesis/ictogenesis and comorbidities, and understanding whether and to what extent psychiatric comorbid conditions are drug-induced and/or drug-aggravated (Fig. 8, right) are hindered by the lack of data on the effects of currently available anti-absence medicines on attention, learning and mood in healthy humans and in normal (young and adult) animals.

### New treatments for absence seizures and their psychiatric comorbid conditions

The pharmaco-resistance of 30% of children with absence seizure, the persistence of neuropsychiatric comorbidities in seizure-free cohorts and the adverse effects of current medications (particularly in polytherapy) demand urgent action on novel treatments. Naturally, the best therapy for any epilepsy is one that would block epileptogenesis, i.e. has a disease-modifying effect. As far as current anti-absence drugs are concerned, some limited evidence in children suggests that early, effective treatment with ethosuximide has positive long-term (≥10 years) outcomes, i.e. seizure- and medication-free (Berg *et al.*, 2014). This potential disease-modifying effect of ethosuximide is strongly supported by results in WAG/Rij and GAERS rats showing that initiating treatment with ethosuximide prior to seizure onset and continuing it for 3–4 months not only fully blocks absence seizures even 3 months after the end of treatment and markedly reduces comorbid anxiety (Blumenfeld *et al.*, 2008; Dezsi *et al.*, 2013), but also increases the mRNA levels of DNA methyltransferases, a key component of epigenetic pathways, in the CIN of GAERS rats (Dezsi *et al.*, 2013) and abolishes the abnormal expression of Na_v_1.1, Na_v_1.6 and HCN1 in WAG/Rij rats (Blumenfeld *et al.*, 2008). In contrast, a shorter treatment (3–5 weeks) initiated even prior to seizure onset does not protect GAERS rats after the end of the treatment (Dedeurwaerdere *et al.*, 2005; Jarre *et al.*, 2017). Indeed, when the start and the duration of the ethosuximide treatment were directly compared in the same study on WAG/Rij rats, only the 4-month treatment (but not the 2-month treatment starting either at the second or fourth month of life) was effective up to 2 months post-treatment (van Luijtelaar *et al.*, 2013). This suggests that either epileptogenic mechanisms are still operant even when SWD appear to be fully developed or that an effective prophylactic treatment needs to affect both epileptogenic and early epileptic processes. Obviously, any potential future translation of an early treatment to humans would require identification of solid genetic (Noebels, 2015*a*; Holmes and Noebels, 2016) or phenotypic biomarkers before the epileptogenic processes have fully established themselves.

As far as novel pharmacological therapies are concerned, there is only one new medication (Epidiolex®), an oral form of cannabidiol, a phytocannabinoid devoid of psychotropic actions (Stockings *et al.*, 2018), that is currently in phase 2 trials in children with pharmaco-resistant absence seizure (ClinicalTrials.gov #NCT03336242; ClinicalTrials.gov #NCT03355300). This is surprising and of concern as this drug was shown to have no effect on non-convulsive absence seizures in previous trials in two severe childhood epilepsies, i.e. Lennox-Gastaux and Dravet syndromes (Devinsky *et al*., 2019; Thiele *et al*., 2018). Moreover, to the best of our knowledge there are no publicly available data on the anti-absence effect of cannabidiol in mice and rats with absence seizures and no study has investigated its potential adverse effects on attention, cognition, learning and memory both in normal non-epileptic young animals and in absence seizure models.

As indicated previously, T-type Ca^2+^ channels of cortical and NRT neurons are key to absence seizure expression (McCafferty *et al.*, 2018*b*) (Fig. 5). This suggests that potent and selective T-channels blockers, in contrast to the weak and non-selective actions of ethosuximide and valproic acid on these channels (Leresche *et al.*, 1998; Crunelli and Leresche, 2002*a*, *b*), may offer concrete hope for pharmaco-resistant absence seizures. Regrettably, the first generation of potent and selective T-channel blockers (Dreyfus *et al.*, 2010) elicited major changes in the vigilance states of absence seizure models (Shipe *et al.*, 2008). However, a member of a new generation of state-dependent T-channel antagonists, CX-8998, is currently in a phase 2 trial in adolescent and adult populations with generalized epilepsy and absence seizures that do not respond to standard of care treatment (ClinicalTrials.gov #NCT03406702, 2018). Moreover, Z944 (now called PRAX-944), another pan-T-channel antagonist that blocks experimental absence seizure and ameliorates some aspects of their comorbidity, is in a phase 2 trial in an adult population with pharmaco-resistant absence seizure (ANZCTR.). It is hoped that CX-8998 and PRAX-944 may, in the near future, be tested for their efficacy and safety in paediatric and juvenile cohorts with absence seizure.

As mentioned earlier, a loss-of-function of GAT-1 in some rat and mouse genetic models leads to increased thalamic GABA levels and to experimental absence seizures via activation of extrasynaptic δ subunit-containing GABA-A receptors (Cope *et al.*, 2009). Thus, drugs that increase the activity of GAT-1 and antagonists of δ-containing GABA-A receptors may represent new targets for treating absence seizure (Errington *et al.*, 2011*a*). Proof-of-concept for this potential therapy is provided by the rescue of the absence phenotype (and lack of adverse effects) in the off-springs of stargazer mice crossed with GABA-A δ-subunit knockout mice (McCafferty *et al.*, 2018*a*). Another interesting target could be the 5-HT2C receptor (Di Giovanni and De Deurwaerdère, 2016), as selective agonists at these receptors have been shown to dose-dependently block experimental absence seizure (Venzi *et al.*, 2016). Finally, positive allosteric modulators of metabotropic glutamate receptors 1 and 5, which are key to cortico-thalamic transmission (Baude *et al.*, 1993; Romano *et al.*, 1995; Errington *et al.*, 2011*c*), block experimental absence seizures (D’Amore *et al.*, 2015; Celli *et al.*, 2019), as does the selective thalamic block of hyperpolarization-activated, cyclic nucleotide-gated channels (David *et al.*, 2018) (see also Hammelmann *et al.*, 2019; Zobeiri *et al.*, 2019), opening up the possibility of selectively targeting one of these receptor/channel subtypes for treating CAE and other epilepsies with absence seizure.

Moving away from pharmaco-therapy, optogenetics has been suggested as a potential future alternative to neurosurgery for adults with pharmaco-resistant epilepsies (Wykes *et al.*, 2012, 2016; Bui *et al.*, 2017; Christenson Wick and Krook-Magnuson, 2018). However, the inherent invasiveness of optogenetic techniques and the substantial incidence of absence seizure remittance in CAE (Camfield and Camfield, 2005) do not lead at present to a favourable risk-benefit analysis for a standard optogenetic therapy. Notably though, the brief reversible and safe opening of the blood–brain barrier induced by non-invasive focused ultrasound in a small brain region (Munoz *et al.*, 2018) allows locally restricted diffusion of an intravenously injected (virally-driven) opsin in mice that can then be activated by transcranial light stimulation (Pouliopoulos *et al.*, 2018). In principle, therefore, following the identification of the CIN in an individual with absence seizures this technique does provide the possibility of delivering and activating light-sensitive proteins without surgical intervention. The safety and reliability of focused ultrasound have already been tested in healthy adults and people with other neurological diseases (Carpentier *et al.*, 2016; Lipsman *et al.*, 2018) but has not been investigated yet in younger populations.

Finally, the block of experimental absence seizures by non-invasive transcranial electrical stimulation (Berenyi *et al.*, 2012; Liu *et al.*, 2018) offers at present a more realistic potential than optogenetics and may have the additional advantage of improving attention as shown in some studies of adult populations (Reteig *et al.*, 2017). However, the implementation of both therapeutic approaches will require improvements of existing algorithms for absence seizure prediction (Maksimenko *et al.*, 2017). Moreover, as transcranial electrical stimulation has been shown to affect brain oscillations and functional connectivity (Krause *et al.*, 2017, 2019) its application to children and teenagers with absence seizures demands knowledge of the long-term effects of repetitive electrical stimulation on brain development before it might be considered a reliable and safe therapeutic avenue.

### Future priorities

#### Mechanism of pre-ictal and abnormal interictal activity

A key future challenge will be to uncover the electrical correlates of the changes in BOLD signal amplitude and synchrony that precede by tens of seconds and even up to 1 min, respectively, the start of absence seizure in humans (Holmes *et al.*, 2004; Bai *et al.*, 2010, 2011; Tangwiriyasakul *et al.*, 2018). Detecting such early pre-ictal changes in EEG spike-wave morphology, frequency, synchrony and/or coupling strength will be fundamental for the development of efficient prediction algorithms (Gadhoumi *et al.*, 2016; Freestone *et al.*, 2017), which in turn are necessary for the potential implementation of transcranial electrical, optogenetic (or other therapeutic/prophylactic) stimulation protocols. So far, no study has systematically analysed the EEG at such early pre-ictal periods in children with absence seizure, and the possibility cannot be discarded that the changes in electrical activity underlying these early pre-ictal functional MRI alterations in humans are preferentially confined to deep cortical layers (Polack *et al.*, 2007) and thus undetectable by standard EEG electrodes.

From an experimental perspective, unambiguous changes in cortical and thalamic single-neuron activity (Fig 4A) (Pinault, 2003; McCafferty *et al.*, 2018*b*; Meyer *et al.*, 2018), in the power of EEG theta, delta and beta frequency bands and in inter-cortical area coupling strengths (Sysoeva *et al.*, 2014, 2016*a*,* b*; Sorokin *et al.*, 2016) have been reported in mouse and rat absence seizure models but they all occur only around 2–4 s prior to seizure onset. As changes in functional MRI BOLD signal are evident up to 1 min before absence seizure onset (Tangwiriyasakul *et al.*, 2018), does this large time difference in early pre-ictal changes represent another key difference between human and animal models because of their potentially different neurovascular coupling? More generally, does it derive from our lack of fully understanding the intricate relationship between electrical and BOLD signals (Goense and Logothetis, 2008), and/or is it a reflection of the fact that BOLD changes in humans with epilepsy and in animal models may not necessarily correlate with, or even relate to, abnormal neuronal activity (Lemieux *et al.*, 2011)?

The early pre-ictal changes of absence seizures are likely to be tightly linked to the presence of a persistently, i.e. interictally, altered cortical sensorimotor network in humans with absence seizures compared to healthy controls (Tangwiriyasakul *et al.*, 2018). Future clinical studies should confirm this finding in larger drug-free cohorts of CAE, whereas the existence and location of similarly abnormal networks during the interictal periods should be investigated in absence seizure models with electrical and/or non-invasive imaging approaches.

#### Mechanism of absence seizure initiation

Whereas the ictogenesis and the temporal dynamics of single-cell interactions underlying cortico-thalamo-cortical network activity in the cycle-by-cycle maintenance of SWDs have started to be unravelled in non-anaesthetized animal models (McCafferty *et al.*, 2018*b*; Meyer *et al.*, 2018), we still lack knowledge of the cellular, synaptic and network mechanisms that lead to absence seizure initiation in the CIN. Because of what has been discussed in the previous point, it is of paramount importance that future studies in animal models must strictly take into account the fact that the dynamic state of single neurons and neuronal networks of the CIN before the start of an electrographic and behavioural absence seizure is not identical to that of the same neurons and networks in a control (i.e. non-epileptic) animal. Moreover, investigations of absence seizure initiation should carefully consider the different location of the CIN among animal models (Meeren *et al.*, 2002; Studer *et al.*, 2018; Lee *et al.*, 2019) and between models and children with absence seizures (Bai *et al.*, 2010; Guo *et al.*, 2016; Tangwiriyasakul *et al.*, 2018).

Understanding the absence seizure initiation process will undoubtedly be a massive and technically highly demanding task that will require an intelligent combination of recording the firing dynamics of identified (or identifiable) neurons from CIN neuronal ensembles in non-anaesthetized animal models coupled with a systematic investigation of subthreshold oscillations and passive and active membrane properties of the various neuronal populations in CIN slices. Some patchy experimental evidence does exist but was obtained in cortical areas other than the CIN or under anaesthetic conditions (D’Arcangelo *et al.*, 2002; Polack *et al.*, 2007; Chipaux *et al.*, 2011). Moreover, while we know that layer 5/6 pyramidal neurons in the CIN of absence models have different intrinsic membrane properties (Kole *et al.*, 2007; Polack *et al.*, 2007) and morphological features (van Luijtelaar and Sitnikova, 2016) than non-epileptic animals, similar information is not available for the different classes of CIN interneurons. Furthermore, there are no data on potential abnormalities of synaptic innervation, strength and plasticity among excitatory and inhibitory neurons in the CIN of any absence seizure model. Indeed, some of the key differences that exist in normal animals between parvalbumin- and somatostatin-positive cortical interneurons (Ascoli *et al.*, 2008) may be altered in the CIN of absence seizure models. Notably, selective inhibition by DREADDS of cortical parvalbumin-positive interneurons has been shown to elicit absence-like seizures in normal mice (Panthi and Leitch, 2019) and genetic ablation of Ca_v_2.1 channels in both parvalbumin- and somatostatin-positive cortical interneurons elicit severe generalized seizures and absence seizures (Rossignol *et al.*, 2013). However, whether and how somatostatin-positive interneurons, the main mediators of the dendritic inhibition-dependent regulation of input-output transformations in pyramidal neurons (Lovett-Barron *et al.*, 2012; Wilson *et al.*, 2012; Lee *et al.*, 2013), may contribute to absence seizure generation remains to be established. Moreover, somatostatin-positive interneurons in the somatosensory cortex are active during quiet wakefulness, the brain state where most absence seizures are expressed (Halász, 2015) and their silencing increases burst firing in cortical pyramidal neurons (Gentet *et al.*, 2012).

#### Mechanism of absence seizure termination

The mechanism underlying the termination of absence seizures has not been directly investigated. Changes in Granger causality values among the activity of cortical and subcortical regions (Lüttjohann *et al.*, 2014) as well as changes in the firing of cortical, thalamic and basal ganglia neuronal populations just around SWD offset have been proposed as potential contributors to absence seizure termination (Paz *et al.*, 2005, 2007; McCafferty *et al.*, 2018*b*) but no causal evidence is available.

From a biophysical perspective, as elegantly summarized by Barthó *et al.* (2014), the length of a SWD (as any other spontaneous transient oscillation) could either be predetermined by the state of the generating network at the start of the SWD or depend on the arrival of a signal (intrinsic or extrinsic to the generating network) at any time after the SWD has started. In the former case, the activity of each SWD follows a fixed trajectory and thus the time of its termination is set by the initial state of the network, whereas in the latter case there is no correlation between the initial and the end state of the SWD-generating network and thus the length (and time of termination) of a SWD. Understanding which of these two scenarios applies to SWD is of paramount importance as it will help to guide more detailed research towards either the key elements that set the conditions of the SWD-generating network at the start of a SWD or the intrinsic and extrinsic perturbations that bring to a stop the activity of the SWD-generating network. Notably, the correlation between EEG-functional MRI data and cognitive impairments during absence seizure in children with CAE has been shown to be already set at the very start of a seizure (Guo *et al.*, 2016).

Because of the hypothesis linking natural sleep spindles (a spontaneous transient oscillation) and SWD of absence seizure (Avoli and Gloor, 1981; Kostopoulos *et al.*, 1981; but see Leresche *et al.*, 2012), currently proposed mechanisms of sleep spindle termination might apply to SWD, though spindles are driven by a thalamic pacemaker with critical cortical modulation (Steriade *et al.*, 1987; Crunelli *et al.*, 2018; Adamantidis *et al.*, 2019; Fernandez and Luthi, 2020) whereas absence seizures require the contribution of many different neuronal populations in cortico-thalamo-cortical and basal ganglia networks. Thus, increased intracellular Ca^2+^ leading to thalamocortical neuron depolarization (Lüthi and McCormick, 1998; Lüthi *et al.*, 1998), desynchronization of the cortical output to the thalamus (Bonjean *et al.*, 2011) and progressive hyperpolarization of NRT neurons (Bal *et al.*, 1995*b*; Kim and McCormick, 1998) coupled with a marked drop in their firing just a few cycles before spindle termination (Barthó *et al.*, 2014), that have been suggested as potential mechanisms for spindle termination, might also be involved in controlling the duration of SWD. Notably, the latter mechanism has been shown to be predetermined by the state of the network at the start of the spindle oscillation in normal (i.e. non-epileptic) rats (Barthó *et al.*, 2014).

Finally, the slowing of the rhythmic activity in both excitatory and inhibitory neurons towards the end of partial (focal) seizures has been suggested to involve a progressive increase in synchrony among participating neurons and to play a role in seizure termination (de Curtis and Avoli, 2015). As a slowing of the SWD frequency is observed within many absence seizure (Fig. 1A) (Panayiotopoulos, 1999; Blumenfeld, 2005*b*; Matricardi *et al.*, 2014) it could be that an increased synchronization of cortical excitatory and inhibitory networks in the CIN may also be critical for absence seizure termination.

#### Mechanism of absence seizure pharmaco-resistance

If we want to prevent the adverse effects of polytherapy, we need to understand the mechanisms underlying pharmaco-resistant absence seizures. As these seizures are genetically determined, a key goal would be to increase efforts in investigating differences in genetic abnormalities between responders and non-responders to currently available anti-absence medications in large cohorts. For experimental research, the priority would be to develop animal models that are insensitive to ethosuximide and valproate. Such a development may be guided by the findings in humans that (i) a more negative treatment outcome, independently of the initial treatment (with either ethosuximide, valproic acid or lamotrigine), occurs for absence seizure without face/limb motor automatisms (Kessler *et al.*, 2017); (ii) ethosuximide responders have decreased parietal cortex connectivity for ictal low frequency EEG components whereas non-responders have increased frontal cortex connectivity for high frequencies (Tenney *et al.*, 2018); and (iii) a *CACNA1H* polymorphism (P640L) is more commonly expressed in children non-responding to ethosuximide monotherapy (Glauser *et al.*, 2017).

#### Mechanism and treatment of absence seizure comorbidities

A major future research task relates to the inability of current therapies to effectively control absence seizure comorbidities since we have no indication as to whether absence seizures and comorbidities share initial common genetic and mechanistic pathways that once fully developed become independent from each other (Sharp *et al.*, 2008; Fejgin *et al.*, 2014; Noebels, 2015*b*; Nilsson *et al.*, 2016). Clinically, the persistence of interictally altered cortical networks (Tangwiriyasakul *et al.*, 2018) calls for routine pre-symptomatic functional MRI and neuropsychiatric analysis for early detection of abnormal cortical network functions in children and teenagers at risk of developing absence seizures. Experimentally, the neuropsychiatric features of absence seizure models should be systematically investigated to understand the underlying neurobiological mechanisms and assess potential treatments. At present, the only drug that has been shown to abolish absence seizures and ameliorate memory deficits in animal models is the pan-T-channel blocker Z944 (Marks *et al.*, 2016*a*; Henbid *et al.*, 2017): selective antagonists for one T-channel subtype may prove more effective than pan T-channel blockers in controlling both absence seizure and associated comorbidity (Powell *et al.*, 2014*a*).

#### Valproic acid-induced comorbidity and continuous neuropsychiatric evaluation

The large incidence of neuropsychiatric comorbidities and the marked increase in attention deficits observed with valproic acid treatment in CAE (Masur *et al.*, 2013) advise against the use of this drug as first monotherapy in children and teenagers with absence seizures. Moreover, the presence of widespread psychiatric comorbidity in individuals with absence seizures makes it of paramount importance to establish closer interactions between neurologists and psychiatrists as part of the standard clinical practice in the initial assessment and continuous follow-up of children and teenagers with CAE, juvenile absence epilepsy and juvenile myoclonic epilepsy. The major efforts that are currently being made towards a better understanding and treatment of attention, cognition and learning deficits and mood disorders (Duman *et al.*, 2016; Postorino *et al.*, 2017; Feldman *et al.*, 2018) will undoubtedly also prove beneficial to children, teenagers and adults with absence seizures.

## Abbreviations

BOLD =: blood oxygenation level-dependent
CAE =: childhood absence epilepsy
CIN =: cortical initiation network
NRT =: nucleus reticularis thalami
SNr =: substantia nigra pars reticulata
SWD =: spike-wave discharges
